# Multiomics profiling reveals that *P. gingivalis*-induced extracellular vesicle reprogramming promotes immune evasion in colorectal cancer through autophagy-mediated STING degradation

**DOI:** 10.3389/fcimb.2025.1604554

**Published:** 2025-10-15

**Authors:** Xu Zhang, Jieying Huang, Guangwei Shang, Yang Lu, Haixia Liu, Yiming Zhang, Fuping Wen, Yuanzhi Xu

**Affiliations:** ^1^ Department of Stomatology, Shanghai Tenth People’s Hospital, Tongji University School of Medicine, Shanghai, China; ^2^ Department of Medical Stomatology, Shanghai Tenth People’s Hospital, Tongji University Cancer Center, Tongji University School of Medicine, Shanghai, China

**Keywords:** *Porphyromonas gingivalis*, colorectal cancer, STING, proteome, miRNA

## Abstract

**Objective:**

*Porphyromonas gingivalis* (*P. gingivalis*) infection is a recognized pathogenic factor in colorectal cancer (CRC), and extracellular vesicles (EVs) are key mediators within the tumor microenvironment. However, the molecular composition of large extracellular vesicles (lEVs) derived from *P. gingivalis*-infected cancer cells remains poorly characterized. This study aimed to comprehensively define the molecular cargo alterations in lEVs secreted by CRC cells in response to *P. gingivalis* infection.

**Methods:**

An integrated multiomics approach was employed to analyze lEVs secreted by *P. gingivalis*-infected HCT116 colorectal cancer cells. miRNA sequencing and quantitative proteomics were used to profile miRNA and protein expression, respectively. Bioinformatic analyses identified differentially expressed molecules. Mechanistic studies involving immunoblotting and autophagy inhibition were conducted to validate and explore key findings.

**Results:**

*P. gingivalis* infection induced significant cargo remodeling in HCT116-derived lEVs. miRNA sequencing identified 223 miRNAs, among which 28 were differentially expressed. Notably, six novel miRNAs were specifically upregulated in lEVs from infected cells. Quantitative proteomics revealed 1,210 significantly altered proteins. Strikingly, 981 proteins were downregulated, including the critical antitumor immune regulator STING (stimulator of interferon genes). STING downregulation in infected HCT116 cells was confirmed, and *P. gingivalis* infection was shown to promote STING degradation via autophagy, explaining its reduced incorporation into lEVs.

**Conclusion:**

This integrated multiomics analysis demonstrates that *P. gingivalis* infection profoundly remodels the molecular landscape of CRC cell-derived lEVs. The specific depletion of immune-stimulating factors, most notably STING, within lEVs suggests a novel mechanism by which this pathobiont may contribute to immune evasion and promote tumor progression in *P. gingivalis*-associated colorectal cancer.

## 1 Introduction

The human microbiome has emerged as a fundamental factor in the complex landscape of cancer biology and is capable of reshaping tumor microenvironments and modulating therapeutic responses across malignancies, including colorectal cancer (CRC) (Saus et al., 2019; Cheng et al., 2020; Cullin et al., 2021; Rahman et al., 2022; Sanidad et al., 2022). Among oncopathogens, *Porphyromonas gingivalis* (*P. gingivalis*), an orally derived bacterium, has been demonstrated to colonize colonic tumors and promote the progression of colorectal cancer (Wang et al., 2021; Kerdreux et al., 2023), leading to poorer patient prognosis (Kerdreux et al., 2023). Beyond its established carcinogenic mechanisms—including induction of a dysbiotic inflammatory microenvironment, inhibition of apoptosis, activation of cell proliferation, enhanced angiogenesis, induction of chronic inflammation, promotion of epithelial-to-mesenchymal transition, and production of carcinogenic metabolites (Lamont et al., 2022)—*P. gingivalis* dysregulates key signaling pathways such as interleukin-1 (IL-1), tumor necrosis factor-α (TNF-α), Notch, MAPK, and NF-κB signaling pathways (Malinowski et al., 2019; Wielento et al., 2022),. Furthermore, *P. gingivalis* upregulates matrix metalloproteinases (MMPs) and IL-8 to promote invasion in oral squamous cell carcinoma (OSCC) (Ha et al., 2016), and is implicated in driving the upregulation of PD-1/PD-L1, often associated with cancers (Liu et al., 2023). It also accelerates CRC progression by inducing CHI3L1 upregulation in invariant natural killer T (iNKT) cells, impairing their cytotoxic functions and promoting tumor immune evasion (Díaz-Basabe et al., 2024). However, the molecular strategies enabling this periodontal pathogen to subvert host antitumor defenses remain poorly understood.

The tumor microenvironment is increasingly recognized as an ecosystem shaped by dynamic interactions between host cells and the resident microbiota (Fu et al., 2024; Zhou et al., 2024). As key mediators of intercellular communication, extracellular vesicles (EVs) transport bioactive molecules, including proteins and miRNAs, that can reprogram recipient cell behavior (Hou and Chen, 2021). Despite their importance, the composition and functions of EVs released from *P. gingivalis*-infected CRC cells remain largely unknown. A comprehensive understanding of these vesicles could provide crucial insights into the mechanisms by which *P. gingivalis* impacts CRC biology.

The intratumoral microbiota, including *Porphyromonas gingivalis* (*P. gingivalis*), is thought to have a profound impact on tumorigenesis and the tumor microenvironment (Iida et al., 2013). One of the key pathways through which it may regulate tumor–host interactions and antitumor immunity is the STING pathway (Zheng and Chen, 2024). STING governs the type I interferon (IFN) signaling cascade, which plays an essential role in initiating innate immune responses. Notably, NLRP3 activation has been found to specifically enhance STING signaling, thereby boosting antitumor immunity even in immunotherapy-resistant CRCs (Mowat et al., 2025). The activation of type I IFN signaling in antigen-presenting cells, such as macrophages and dendritic cells, is vital for inducing tumor-specific adaptive immune responses, including the activation of cytotoxic CD8+ T cells (Chin et al., 2023). In the tumor microenvironment, extracellular vesicle-delivered STING can be taken up by immune cells, potentially triggering a series of events that culminate in antitumor immune responses (Jang et al., 2021; McAndrews et al., 2021; Gao et al., 2022). However, microbial pathogens are increasingly recognized to manipulate STING signaling as an immune evasion strategy. For instance, certain DNA viruses induce STING degradation to facilitate viral persistence and replication (Bai et al., 2023; Tao et al., 2023). This evidence raises a pivotal question: Could *P. gingivalis* compromise host defenses through EV-mediated STING suppression?

In this study, we employed multiomics profiling to determine the molecular consequences of *P. gingivalis* infection on large extracellular vesicles (lEVs) derived from CRC cells—an area that remains relatively unexplored. Our integrated proteomic and miRNA analyses revealed infection-specific cargo remodeling characterized by STING depletion in lEVs. Mechanistically, we demonstrated that *P. gingivalis* induces the autophagic degradation of STING in HCT116 cells, effectively preventing its incorporation into EVs. These findings reveal that *P. gingivalis* subverts antitumor immunity through STING pathway inhibition via extracellular vesicle-mediated communication, elucidating a novel mechanism of microbiota-driven immune evasion in colorectal cancer. Furthermore, this study establishes a comprehensive miRNA and proteomic database characterizing EV-carried bioactive molecules remodeled by *P. gingivalis* infection. This valuable resource provides critical insights into how *P. gingivalis* reshapes the tumor microenvironment through vesicular cargo reprogramming.

## 2 Results

### 2.1 *P. gingivalis* infection and lEV identification

**Figure 1:**
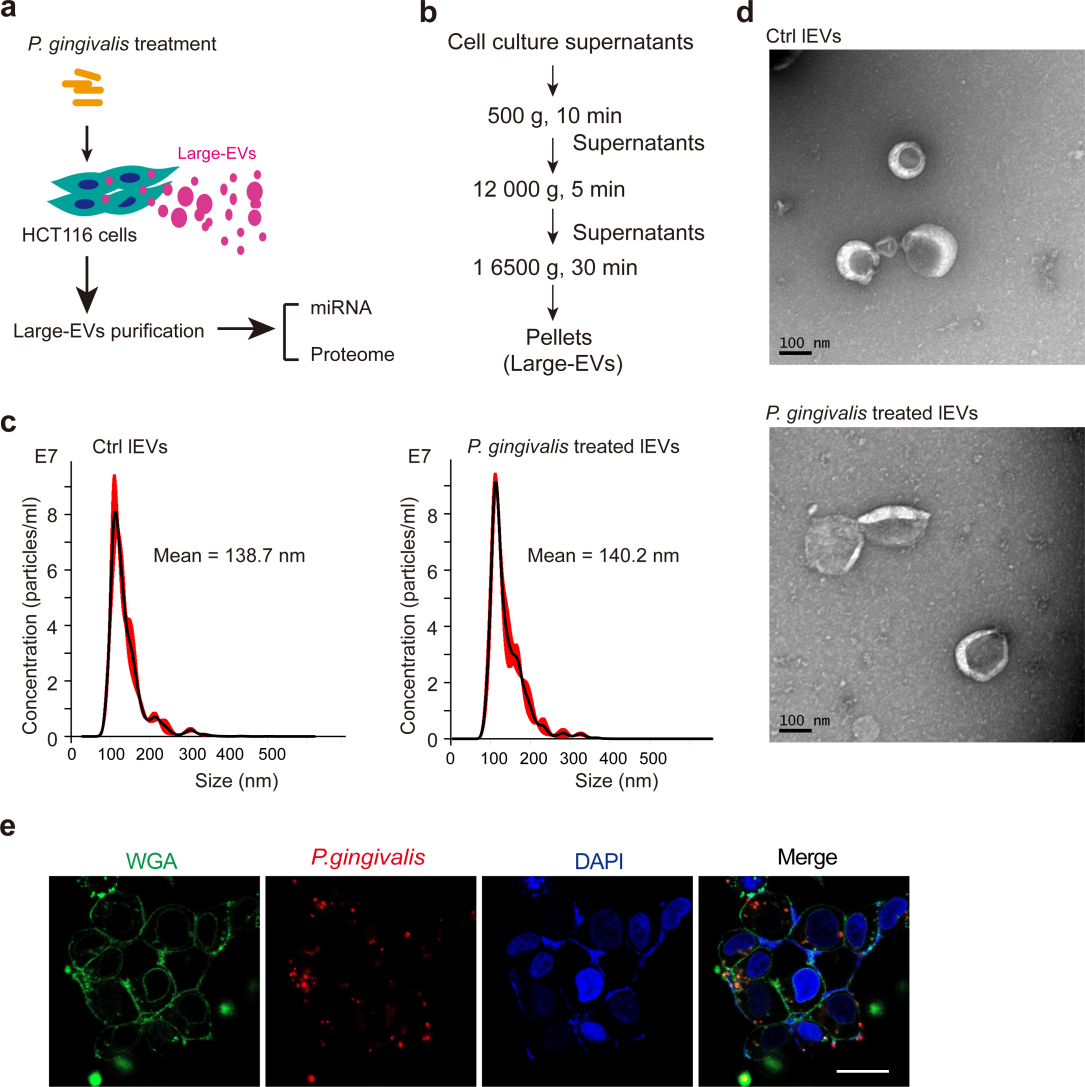
Purification of lEVs from *P. gingivalis*-infected HCT116 cells. **(a)** Schematic showing the generation and mass spectrometry identification of large extracellular vesicles (lEVs) from *P. gingivalis*–infected HCT116 cells. *P. gingivalis* at a multiplicity of infection (MOI) of 100 was used for treatment. **(b)** Centrifugation protocol and workflow for lEV enrichment from *P. gingivalis*–infected HCT116 cells. **(c)** Representative particle size distribution of lEVs from *P. gingivalis*–infected or uninfected HCT116 cells determined by nanoparticle tracking analysis. Three biological replicates were analyzed for each group. **(d)** Transmission electron microscopy (TEM) image of lEVs isolated from *P. gingivalis*–infected or uninfected HCT116 cells. Scale bar = 100 nm. **(e)** Visualization of *P. gingivalis* infection in HCT116 cells. Bacteria were pre-stained with eFluor™ 670 (red), followed by infection of HCT116 cells. After 12 h, cell membranes were labeled with wheat germ agglutinin (WGA; green), and nuclei were counterstained with DAPI (blue).

To comprehensively identify the bioactive components of large extracellular vesicles (lEVs) derived from colorectal cancer cells infected with *Porphyromonas gingivalis* (*P. gingivalis*), HCT116 cells were infected with *P. gingivalis* at a multiplicity of infection (MOI) of 100 (**Figure 1a**). Subsequently, lEVs were isolated via a series of differential centrifugation steps (**Figure 1b**). The purified lEVs from control or *P. gingivalis-*infected HCT116 cells were then subjected to nanoparticle tracking analysis (NTA) and transmission electron microscopy (TEM) (**Figures 1c, d**). Additionally, immunofluorescence analysis confirmed that *P. gingivalis* effectively infected HCT116 cells (**Figure 1e**). The purified lEVs exhibited the expected size range (100 nm to 1 μm) and characteristic lipid-bilayered morphology, confirming their suitability for subsequent miRNA sequencing and label-free quantitative proteomic analyses.

### 2.2 miRNA sequencing of *P. gingivalis*-infected HCT116 cells

**Figure 2:**
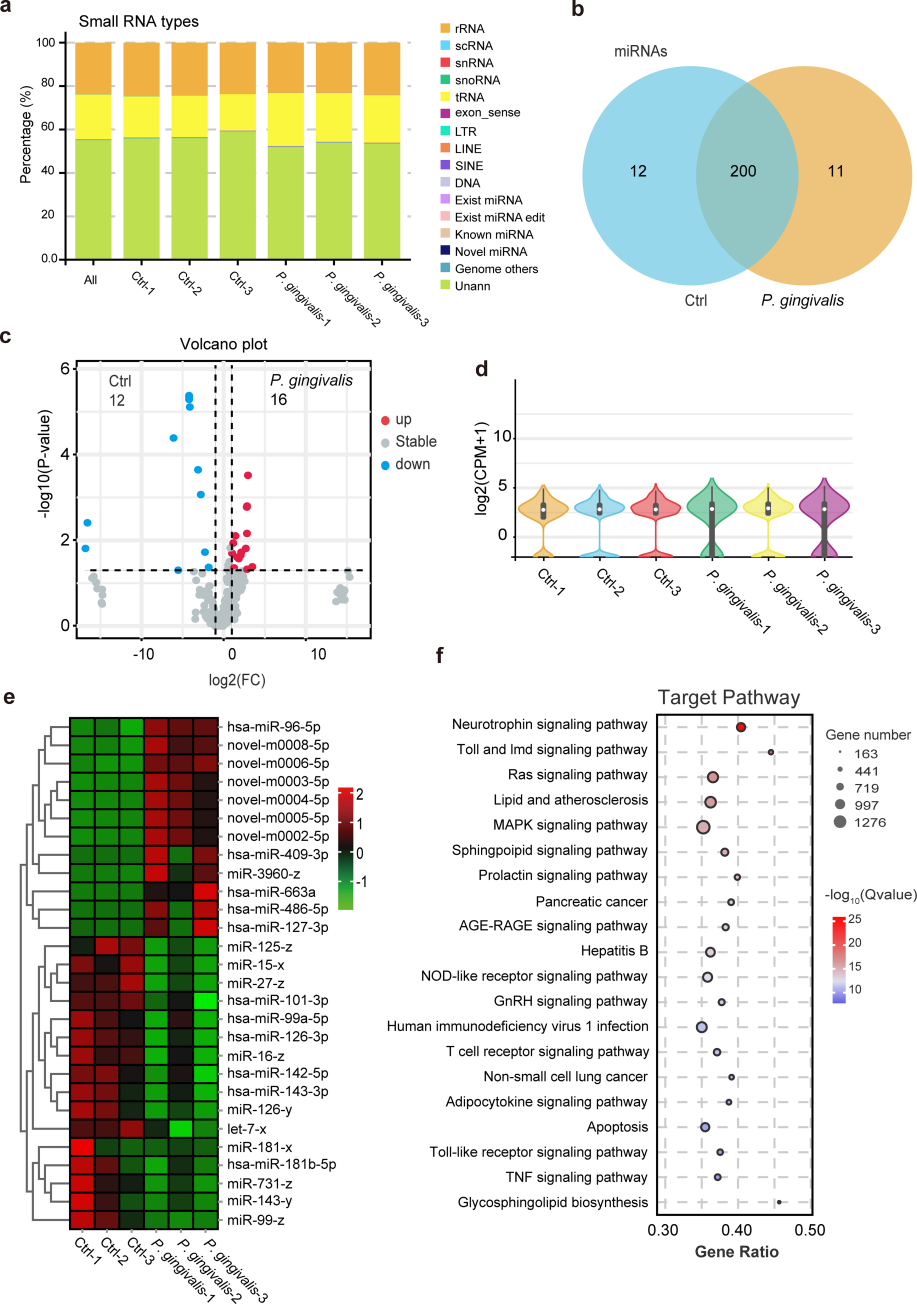
miRNA profile of lEVs from *P. gingivalis*-infected HCT116 cells. **(a)** Bar plot showing the distribution of small RNA types in lEVs derived from *P. gingivalis*–infected or uninfected HCT116 cells. Three biological replicates were used per group. **(b)** Venn diagram showing the overlap between miRNAs in control and *P. gingivalis*–infected HCT116 lEVs. **(c)** Volcano plot showing differentially expressed miRNAs in lEVs derived from control and *P. gingivalis*–infected cells. **(d)** Violin plot showing the expression levels of identified miRNAs in each sample. **(e)** Heatmap showing quantification of differentially expressed miRNAs. Data represent three biological replicates. **(f)** Kyoto Encyclopedia of Genes and Genomes (KEGG) pathway analysis showing the enrichment of differentially expressed miRNAs in the lEVs of *P. gingivalis*–infected HCT116 cells. Data visualization was performed using R packages.

To systematically characterize *P. gingivalis* infection–specific EV cargo profiles, we isolated lEVs from HCT116 colorectal cancer cells with or without *P. gingivalis* infection. Total RNA was extracted from three biological replicates per group, separated via agarose gel electrophoresis, and fragments of 18–30 nucleotides (small RNAs) were excised for high-throughput sequencing. High-throughput sequencing generated 27,849,148 total reads, most of which were derived from tRNA (20.57%), rRNA (23.84%), and unknown RNA (55.06%) molecules (**Figure 2a**). Through miRBase alignment and novel miRNA prediction, a total of 223 miRNAs were identified (**Figure 2b**; **Supplementary Table S1**), with only 0.09% (24,514 reads) of the clean reads mapped to known miRNA sequences.

Comparative bioinformatic analysis revealed significant alterations in lEV miRNA profiles following *P. gingivalis* infection: 16 miRNAs were upregulated and 12 were downregulated in lEVs derived from infected HCT116 cells (**Figure 2c**; **Supplementary Table S1**). A violin plot revealed conserved expression patterns for most miRNAs, although six novel miRNAs (not annotated in current databases) were exclusively detected in infection-associated EVs (**Figures 2d, e**). Functional enrichment analysis mapped these differentially expressed miRNAs to critical oncogenic signaling cascades (including the Ras/MAPK signaling pathway), immune regulation (the Toll-like receptor signaling pathway, NOD-like receptor signaling pathway, and TNF signaling pathway), and immune-metabolic processes such as lipid metabolism and atherosclerosis regulation (**Figure 2f**).

### 2.3 Proteome profile of lEVs from *P. gingivalis*-infected HCT116 cells

**Figure 3:**
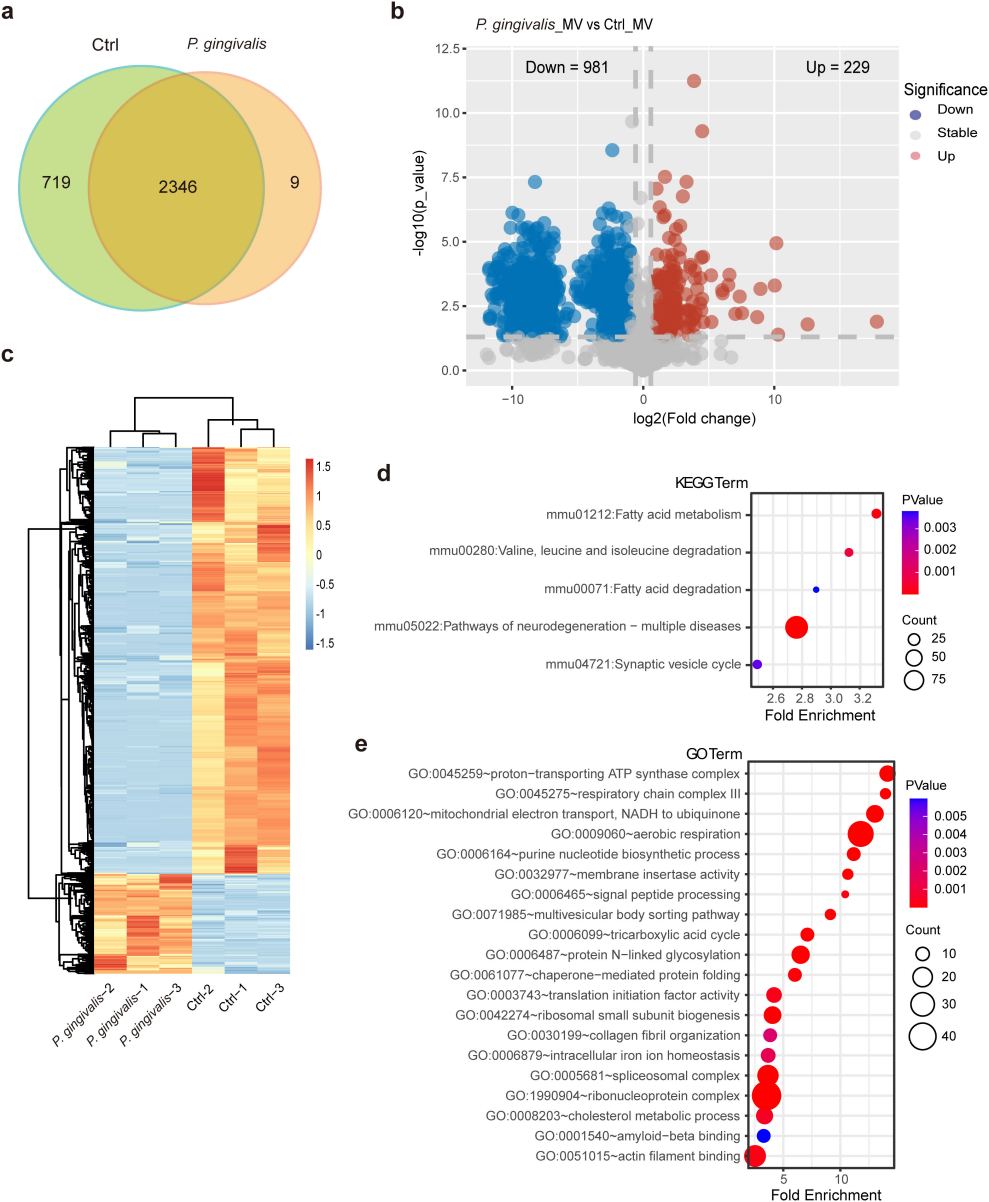
Proteomic analysis of lEVs from *P. gingivalis*-infected HCT116 cells. **(a)** Venn diagram showing the overlap between proteins in uninfected and *P. gingivalis*–infected HCT116 lEVs. **(b)** Volcano plot showing differentially expressed proteins in lEVs from control and *P. gingivalis*–infected cells. Blue dots indicate proteins downregulated by infection, and red dots indicate proteins upregulated. **(c)** Heatmap showing quantification of differentially expressed proteins. Data represent three biological replicates. **(d, e)** KEGG and Gene Ontology (GO) enrichment analyses showing pathways associated with differentially regulated proteins in the lEVs of *P. gingivalis*–infected HCT116 cells. Circle diameter indicates the number of genes, color represents *p* values, and fold enrichment indicates the level of enrichment for a given GO term.

Studies have revealed microbial compositional and ecological alterations in patients with colorectal cancer (CRC) (Lynch and Pedersen, 2016; White and Sears, 2024). These microbes influence multiple aspects of CRC, including pathogenesis, the tumor microenvironment (TME), immune responses, extracellular vesicle secretion, and the effectiveness of therapeutic interventions for CRC (Wong and Yu, 2023; Xia et al., 2023). To identify the proteomic changes in large extracellular vesicles (lEVs) directly induced by *Porphyromonas gingivalis* (*P. gingivalis*) infection, we applied quantitative proteomics to analyze proteome alterations in purified lEVs. A total of 3,074 proteins were identified (**Figure 3a**; **Supplementary Table S2**), among which 1,210 exhibited more than a twofold change between *P. gingivalis*–infected and noninfected lEV samples (**Figures 3b, c**; **Supplementary Table S3**). Of these differentially regulated proteins, 981 were downregulated and 229 were upregulated in lEVs generated from *P. gingivalis*–infected HCT116 cells (**Figure 3c**). Integrated Kyoto Encyclopedia of Genes and Genomes (KEGG) pathway enrichment and Gene Ontology (GO) analyses revealed that the differentially expressed proteins were predominantly associated with fatty acid metabolism; pathways of neurodegeneration—multiple diseases; energy metabolism processes (including the proton-transporting ATP synthase complex, tricarboxylic acid cycle, respiratory chain complex III, and aerobic respiration); and translational regulation processes (such as ribosomal small subunit biogenesis, spliceosomal complex, and ribonucleoprotein complex), as illustrated in **Figures 3d, e**. These findings demonstrate that *P. gingivalis*–induced modification of lEVs derived from colorectal cancer cells may remodel metabolic networks and maintain energy homeostasis within the TME.

Notably, proteomic profiling revealed specific expression of stimulator of interferon genes (STING1) in uninfected lEVs. STING plays a crucial role in mediating antitumor immunity (Jang et al., 2021; McAndrews et al., 2021; Luo et al., 2024) through type I interferon signaling and immune cell activation. The depletion of this key immune regulatory protein in tumor-derived lEVs suggests a potential mechanism for immune evasion.

### 2.4 STING degradation via autophagy in *P. gingivalis*-infected HCT116 cells

**Figure 4:**
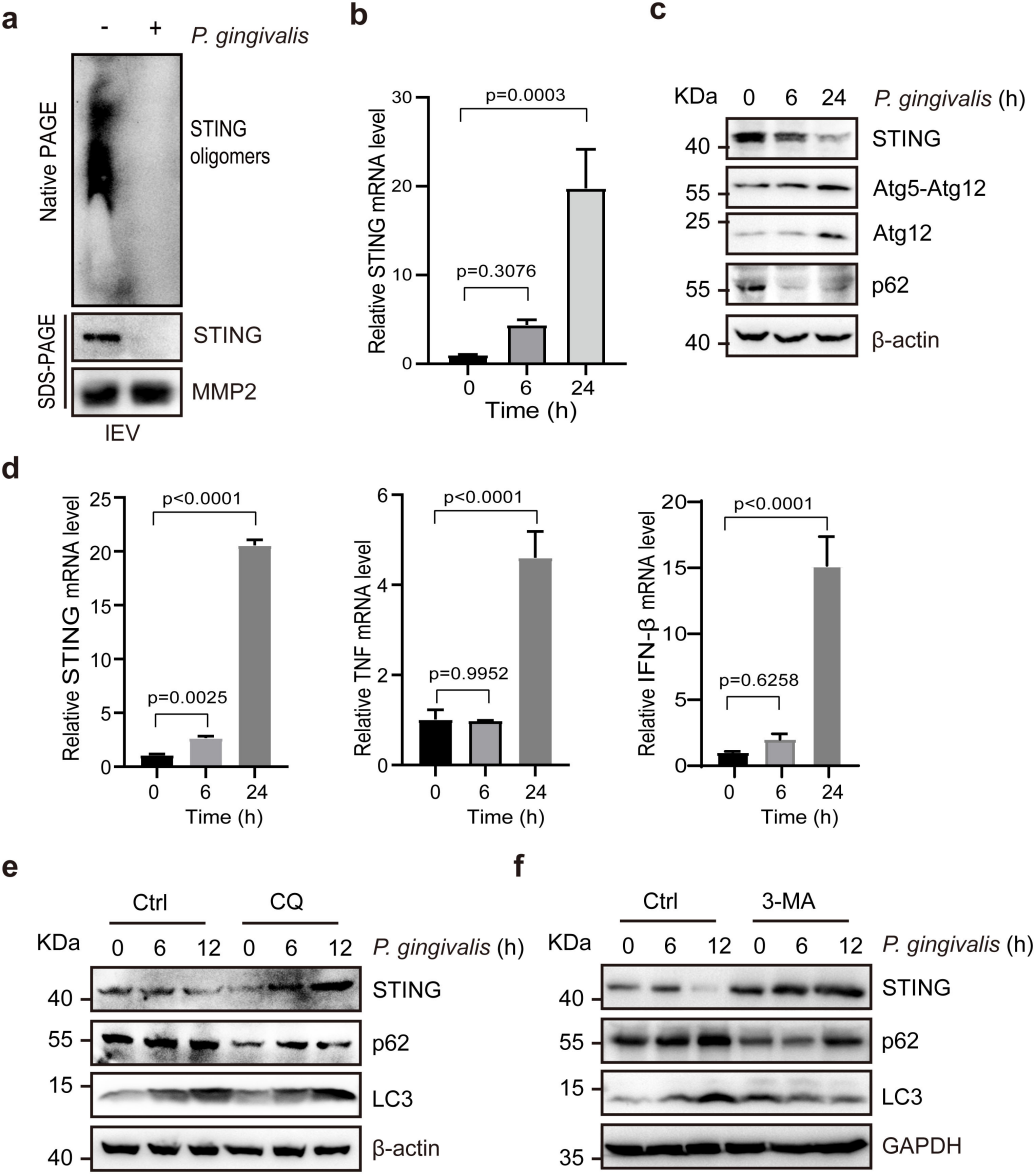
P*. gingivalis* infection induced autophagy and degraded STING in HCT116 cells. **(a)** Immunoblotting showing that the number of extracellular vesicles containing STING from HCT116 cells was significantly reduced after *P. gingivalis* infection. HCT116 cells were infected with *P. gingivalis* (MOI 100) for 48 h, and supernatants were used for lEV purification and Western blotting. **(b)** mRNA levels of STING in HCT116 cells after 0, 6, and 24 h of infection with *P. gingivalis* (MOI 100). Three replicates were analyzed per time point. Statistical analysis and data visualization were performed using GraphPad Prism 9 software. **(c)** Immunoblotting showing that STING expression was significantly decreased, while autophagy was induced after *P. gingivalis* infection. HCT116 cells were treated with *P. gingivalis* (MOI 100) for 0, 6, or 24 h. **(d)** Time-dependent changes in mRNA levels of STING, TNF, and IFN-β following *P. gingivalis* infection (MOI 100) at 0, 6, and 24 h. **(e)** Immunoblotting showing that STING expression was rescued when autophagy was inhibited with 50 µM chloroquine. HCT116 cells were treated with or without *P. gingivalis* (MOI 100) for 0, 6, or 12 h. **(f)** Western blot showing STING, LC3, and p62 expression in control and autophagy-inhibited cells under *P. gingivalis* infection. 3-Methyladenine (3-MA; 5 mM) was used to inhibit autophagy. HCT116 cells were treated with or without *P. gingivalis* (MOI 100) for 0, 6, or 12 h.

STING has been reported to be secreted by extracellular vesicles in oligomeric form (Liang and Yin, 2023), and here we confirmed that STING can indeed be secreted by lEVs and that *P. gingivalis* infection significantly reduces the secretion of STING by lEVs, as shown by Western blotting (**Figure 4a**). The downregulation of STING in lEVs could result from changes in the mRNA or protein expression levels of STING in cells, or from a blockade of STING secretion into lEVs. Therefore, we examined the mRNA and protein levels of STING in *P. gingivalis*–infected HCT116 cells. Quantitative PCR results revealed that STING mRNA levels increased (**Figure 4b**), whereas Western blot analysis indicated that STING protein levels were significantly reduced in response to *P. gingivalis* infection (**Figure 4c**). These results suggest that although STING transcription increased, the expressed protein was rapidly degraded after *P. gingivalis* infection.

Autophagy has been reported to play a crucial role in regulating and fine-tuning cGAS–STING signaling (Gonugunta et al., 2017; Schmid et al., 2024). Conversely, the STING pathway can induce both canonical and noncanonical forms of autophagy (Gui et al., 2019; Zhang et al., 2021; Wan et al., 2023; Schmid et al., 2024). Here, the expression of the ATG12–ATG5 complex, which is essential for autophagy, was significantly increased, and the protein expression of the selective autophagy marker p62 was significantly decreased after *P. gingivalis* infection (**Figure 4c**), suggesting that autophagic activity was significantly increased after *P. gingivalis* infection, as previously reported (Liu et al., 2018; Zhao et al., 2025). We also measured the mRNA expression levels of TNF-α and IFN-β (target genes regulated by STING signaling) via quantitative PCR. The results revealed that the mRNA levels of TNF-α and IFN-β were increased at an MOI of 100 in *P. gingivalis*–infected cells (**Figure 4d**). To further elucidate the relationship between STING and autophagy in CRC, we infected HCT116 cells with *P. gingivalis* while blocking autophagy with chloroquine (CQ). Western blot analysis revealed a significant increase in STING levels upon autophagy inhibition by CQ (**Figure 4e**). Similarly, inhibition of autophagy with 3-methyladenine (3-MA), a class III phosphatidylinositol 3-kinase (PI3K) inhibitor, blocked *P. gingivalis*–induced autophagy and prevented STING degradation (**Figure 4f**). These results suggest that *P. gingivalis* infection induces both STING signaling and autophagy, but the STING protein is degraded by activated autophagy.

### 2.5 *P. gingivalis* infection regulated STING-interacting proteins

**Figure 5:**
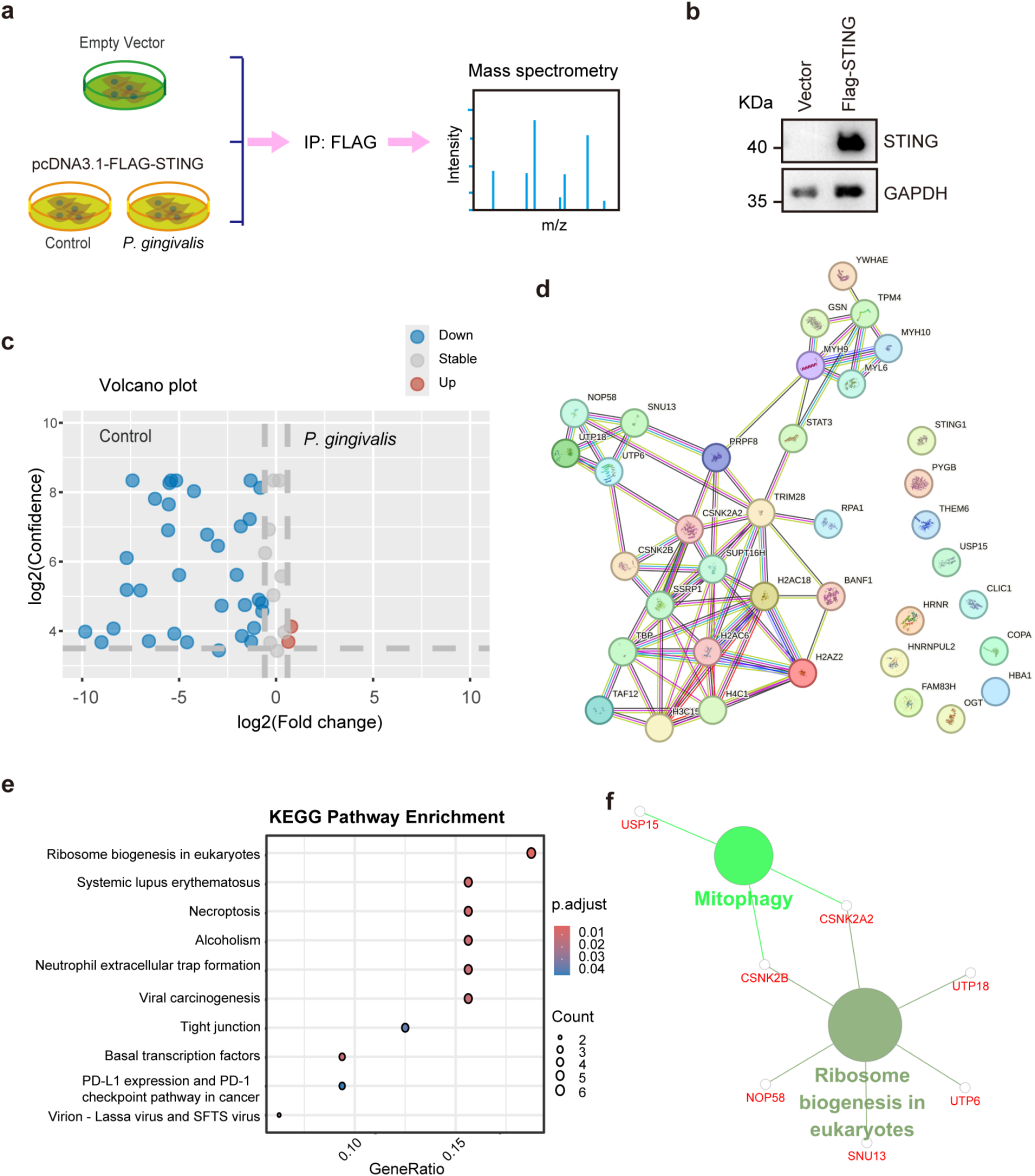
P*. gingivalis* infection remodels the STING interactome in CRC. **(a)** Diagram showing the workflow for identifying STING-interacting proteins affected by *P. gingivalis* infection. **(b)** Validation of FLAG-STING expression in HCT116 cells via Western blotting. **(c)** Volcano plot showing changes in STING-interacting proteins after *P. gingivalis* infection in HCT116 cells. **(d)** Protein–protein interaction (PPI) network analysis of STING-associated proteins whose expression changed after infection. The analysis was performed using the STRING online database (https://cn.string-db.org/). **(e, f)**. Functional consequences of STING interactome remodeling. KEGG analysis showing pathway enrichment of differentially regulated STING-interacting proteins in response to *P. gingivalis* infection. The KEGG analysis was performed using RStudio **(e)** and the Cytoscape software 3.8.2 ClueGO plugin **(f)**.

To investigate the regulatory effects of *Porphyromonas gingivalis* (*P. gingivalis*) infection on STING-interacting proteins and the functional consequences of STING degradation in colorectal cancer cells, we transfected pcDNA3.1-FLAG-STING into HCT116 cells and treated them with or without *P. gingivalis* infection for 48 h. Subsequently, STING-interacting proteins were coimmunoprecipitated using anti-FLAG magnetic beads, and the precipitated proteins were subjected to mass spectrometry analysis (**Figure 5a**). HCT116 cells transfected with the empty vector served as a negative control. Successful FLAG-STING expression was confirmed by Western blot analysis (**Figure 5b**). Mass spectrometry analysis revealed that 46 proteins specifically interacted with STING, each showing a >5-fold change relative to the control. During *P. gingivalis* infection, two proteins exhibited significantly enhanced binding affinities, whereas 34 displayed markedly diminished associations with STING (**Figure 5c**; **Supplementary Table S4**). Subsequent protein–protein interaction (PPI) network analysis using the STRING database demonstrated that 26 of these differentially associated proteins had established interactions (**Figure 5d**). KEGG pathway enrichment analysis revealed that STING-interacting proteins during *P. gingivalis* infection were predominantly enriched in ribosome biogenesis, systemic lupus erythematosus, necroptosis, neutrophil extracellular trap formation, and the PD-L1/PD-1 checkpoint pathway in cancer (**Figure 5e**). ClueGO functional network analysis further demonstrated that these STING-interacting proteins mainly participated in mitophagy and ribosome biogenesis (**Figure 5f**), consistent with previous studies documenting crosstalk between STING and autophagy (Schmid et al., 2024; Zhou et al., 2024). Notably, these pathway alterations, together with the autophagic degradation of STING, suggest that *P. gingivalis*–induced STING elimination may fundamentally remodel the immune landscape of colorectal cancer cells. Collectively, these findings indicate that *P. gingivalis* can mechanistically facilitate immune evasion in colorectal cancer (CRC) through targeted modulation of immune surveillance pathways and checkpoint molecule expression.

## 3 Discussion

**Figure 6:**
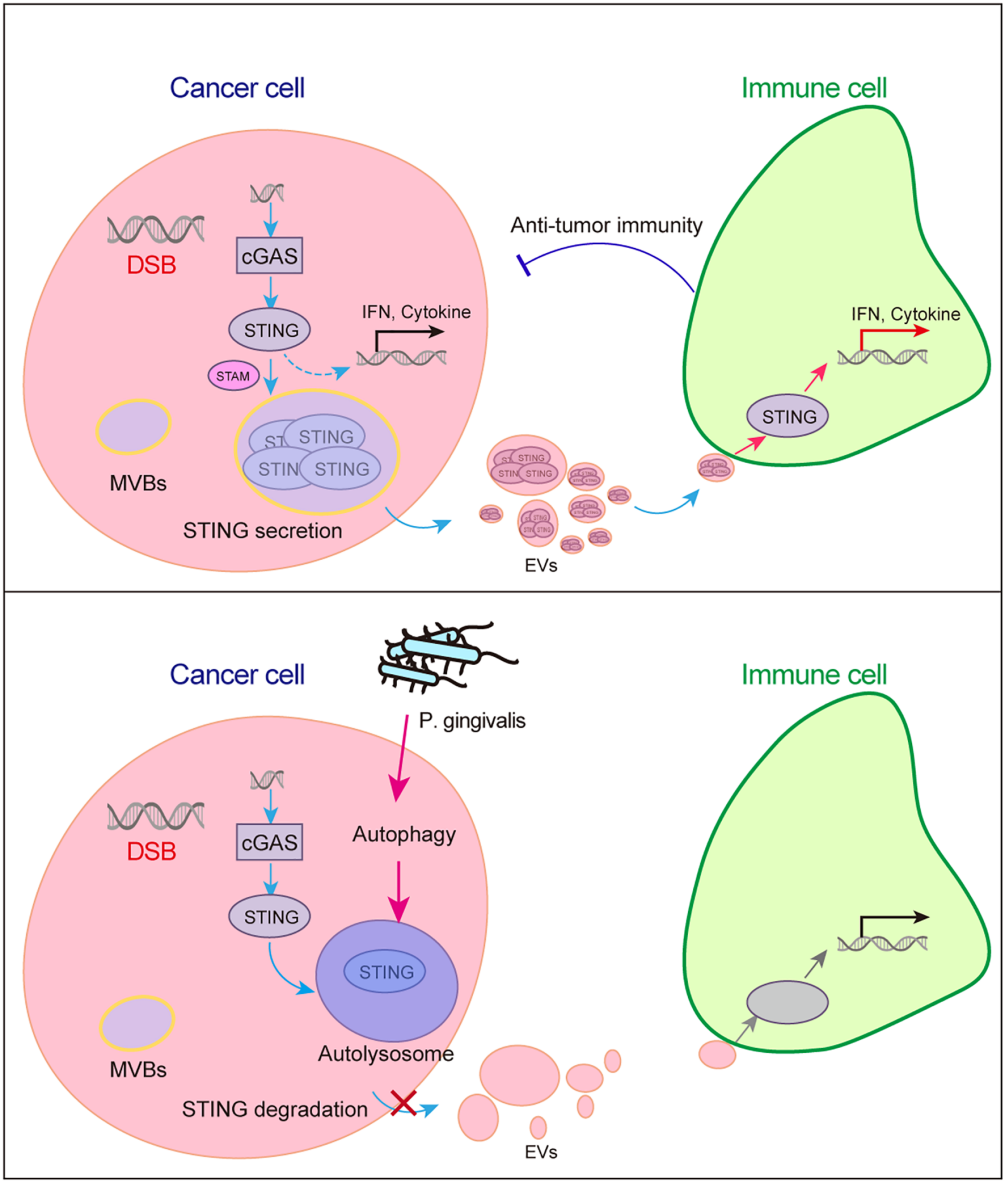
P*. gingivalis* infection *confers resistance* to STING-induced antitumor immunity. Radiation induces DNA double-strand breaks (DSBs), STING activation, and antitumor immunity in cancer cells (upper panel). However, when cancer cells are infected with *P. gingivalis*, STING is degraded via autophagy, thereby suppressing STING-mediated antitumor immunity (lower panel).

Colorectal cancer (CRC) is the third most prevalent malignancy and the second leading cause of cancer-related death worldwide (Bray et al., 2024). The microbiota plays a crucial role in CRC progression and therapeutic efficacy. Recent studies have indicated that *P. gingivalis* infection is a significant contributor to CRC; however, its influence on the tumor microenvironment (TME) and antitumor immunity remains unclear. This study provides compelling evidence that *P. gingivalis* infection remodels the molecular cargo of CRC-derived large extracellular vesicles (lEVs), with profound implications for immune evasion (**Figure 6**). Through integrated miRNA and proteomic profiling, we revealed that *P. gingivalis* infection induces the selective depletion of immunostimulatory molecules—most notably STING—while enriching oncogenic miRNAs and metabolic regulators in lEVs. These findings position *P. gingivalis* as a microbial orchestrator of TME reprogramming through vesicle-mediated intercellular communication.

The autophagy-mediated STING degradation observed here provides a mechanistic link between *P. gingivalis* infection and impaired antitumor immunity. Our multiomics profiling also revealed coordinated EV alterations beyond STING. However, our results contrast with reports that *P. gingivalis* upregulates STING in oral fibroblasts to induce inflammation (Bi et al., 2023). This discrepancy may reflect tissue-specific regulation, underscoring the need to evaluate microbial effects across different cancer types. Furthermore, the downregulation of mitochondrial respiratory proteins observed here suggests a microbial strategy to reprogram tumor energy metabolism, potentially favoring glycolytic pathways over oxidative phosphorylation. The six novel miRNAs identified in this study may also represent unique therapeutic targets for *P. gingivalis*–associated CRC.

Notably, autophagy and STING signaling exhibit bidirectional regulatory interactions (Gonugunta et al., 2017; Zhang et al., 2021). In this study, *P. gingivalis* infection initially induced STING expression, followed by its rapid degradation via autophagy. This phenomenon may explain the clinical observations of chronic inflammation coexisting with immune evasion (Hayashi et al., 2010; Zheng et al., 2021) in *P. gingivalis*–associated CRC. Moreover, STING interactome analysis revealed concomitant PD-L1 pathway modulation, suggesting bacterial potentiation of immune checkpoint resistance. This finding aligns with previous evidence of *P. gingivalis*–mediated PD-1 pathway regulation (Liu et al., 2023), though further validation is warranted.

The limitations of this study are as follows. First, we focused on HCT116 cells; validation in primary CRC organoids and *in vivo* models is needed to confirm physiological relevance. Furthermore, analysis of the correlation between *Porphyromonas* gingivalis (*P. gingivalis*) colonization and EV-STING levels in CRC patients was precluded by the available data. Such an investigation would add significant value and guide future research. Second, although we identified autophagy as the mechanism underlying STING degradation, the upstream triggers (e.g., bacterial virulence factors) remain uncharacterized. It has been reported that lipopolysaccharide (LPS) from *P. gingivalis* promotes autophagy via the PI3K/Akt/mTOR signaling pathway in cells (Liu et al., 2018; Lu et al., 2024); however, we did not confirm whether autophagy is stimulated by *P. gingivalis* LPS or by other bacterial components in CRC. Third, the immunosuppressive effects of modified extracellular vesicles require functional validation using coculture systems with immune cells. Fourth, although strain-specific differences were not examined in this study, future work should validate these mechanisms in clinical isolates and additional *P. gingivalis* reference strains (e.g., W83). Finally, clinical correlations between *P. gingivalis* colonization, EV-STING levels, and patient survival should be explored.

Overall, our integrated analysis identifies *P. gingivalis* as a master regulator of extracellular vesicle–mediated immune evasion in CRC. By coupling STING degradation with oncogenic miRNA enrichment, this pathogen may synergistically disable host defenses and promote tumor progression. Future studies targeting the STING or autophagy pathways could counteract microbially driven immunosuppression, offering new avenues for microbiome-informed CRC therapies.

## 4 Materials and methods

### 4.1 Cell culture and treatment

The colorectal cancer cell line HCT116 was purchased from the American Type Culture Collection (ATCC, CCL-247, USA) and cultured in McCoy’s 5A medium supplemented with 10% fetal bovine serum (FBS), 1% glutamine, 1% sodium pyruvate, and 1% penicillin–streptomycin. The cells were maintained in an incubator at 37°C with 5% CO_2_. For bacterial treatment, HCT116 cells were cocultured with *Porphyromonas gingivalis* (*P. gingivalis*) at the indicated multiplicity of infection (MOI).

### 4.2 Bacterial culture and infection

*P. gingivalis* (ATCC33277) was cultured and prepared for experiments as previously described (Angabo et al., 2024). In brief, *P. gingivalis* was grown in brain heart infusion (BHI) broth supplemented with 0.25% yeast extract, 5 µg/mL hemin, and 1 µg/mL vitamin K under anaerobic conditions (anaerobic chamber) at 37 °C without shaking. Bacteria were collected at an optical density (OD) of approximately 0.4 and centrifuged at 1,500 × g. After being washed three times with phosphate-buffered saline (PBS), *P. gingivalis* was resuspended in PBS, and the bacterial concentration was determined microscopically. HCT116 cells were infected with *P. gingivalis* at an MOI of 100. Bacteria and cells were then cocultured in medium for the indicated durations.

To verify infection efficiency, *P. gingivalis* was stained with Cell Proliferation Dye eFluor™ 670 (Thermo Fisher Scientific, 65-0840-85) according to the manufacturer’s instructions for 30 min. The stained bacteria were then washed with PBS and used to infect HCT116 cells. After 12 h of infection, cells were washed with PBS, and the plasma membrane was labeled with wheat germ agglutinin (WGA; Thermo Fisher Scientific, W11261). Nuclei were stained with 4′,6-diamidino-2-phenylindole (DAPI).

### 4.3 Extracellular vesicle purification

The isolation and purification of extracellular vesicles (EVs) were conducted as previously described (Wen et al., 2024). In brief, HCT116 cells were cultured in Dulbecco’s modified Eagle’s medium (DMEM) supplemented with 10% exosome-depleted FBS. The cell culture supernatants were collected and centrifuged at 500 × g for 10 min, followed by 12,500 × g for 5 min to remove cell debris and dead cells. Large extracellular vesicles (lEVs) were pelleted by centrifugation at 16,500 × g for 45 min and resuspended in cold PBS. All centrifugation steps were performed at 4°C. The isolated lEVs were stored at −80 °C until further use.

### 4.4 NTA analysis

Nanoparticle tracking analysis (NTA) was performed as previously described (Wen et al., 2024). In brief, lEVs from HCT116 cells were purified by differential centrifugation, and the nanoparticle concentration and size distribution of lEVs were measured using a NanoSight NS300 instrument (Malvern). The NanoSight system was equipped with a 488 nm blue laser and a high-sensitivity sCMOS camera. During the measurements, the temperature was maintained at 25°C. The average values from three biological replicates were used to determine the mode size distribution and particle concentration.

### 4.5 TEM analysis

To verify the presence of purified extracellular vesicles (EVs) via transmission electron microscopy, purified large extracellular vesicles (lEVs) were suspended in cold phosphate-buffered saline (PBS) and dropped onto Formvar carbon-coated nickel grids. After 1 min, the grids were washed three times with PBS and stained with 2% uranyl acetate for 1 min at room temperature. Excess stain was removed with filter paper, and the grids were air-dried and visualized using a Tecnai G2 Spirit 120 kV transmission electron microscope (FEI).

### 4.6 RNA extraction and quantitative PCR

Total RNA was extracted from HCT116 cells using an RNA isolate kit (R401, Vazyme) according to the manufacturer’s instructions. One microgram of total RNA from each sample was reverse-transcribed into cDNA using a First-Strand cDNA Synthesis Kit (11141ES60, Yeasen). Quantitative PCR (qPCR) was performed using SYBR Green Master Mix (11201ES03, Yeasen), with GAPDH serving as the internal control.

The following primer sequences were used: GAPDH (primer F: GGAGCGAGATCCCTCCAAAAT, primer R: GGCTGTTGTCATACTTCTCATGG), IFN-β (primer F: GCTTGGATTCCTACAAAGAAGCA, primer R: ATAGATGGTCAATGCGGCGTC), STING (primer F: CCAGAGCACACTCTCCGGTA, primer R: CGCATTTGGGAGGGAGTAGTA), and TNF (primer F: CCTCTCTCTAATCAGCCCTCTG, primer R: GAGGACCTGGGAGTAGATGAG).

### 4.7 Western blotting and antibodies

lEVs or cell pellets were lysed in RIPA buffer (50 mM Tris-HCl, pH 7.4; 150 mM NaCl; 0.1% SDS; 5 mM EDTA; 2 mM sodium pyrophosphate; 25 mM β-glycerophosphate; 1% Triton X-100; 10 mM NaF; 0.5 mM DTT; 1 mM PMSF; and a protease inhibitor cocktail) on ice. The lysate was centrifuged at 13,500 × g for 30 min, and the supernatant was used for protein quantification via a BCA protein assay kit. Samples were mixed with loading buffer, boiled for 10 min, separated by 10% SDS–PAGE, and transferred onto nitrocellulose membranes. Membranes were blocked with 5% nonfat milk for 1 h and then incubated with primary antibodies overnight at 4°C. After three PBS washes, membranes were incubated with secondary antibodies for 1 h, developed using enhanced chemiluminescence (ECL) reagents (36208ES76, Yeasen Biotechnology), and visualized using an enhanced chemiluminescence imaging system.

The following primary antibodies were used: anti-STING (13647, Cell Signaling Technology); anti-MMP2 (ab92536, Abcam); anti-ATG5 rabbit mAb (12994, Cell Signaling Technology); anti-ATG12 rabbit mAb (4180, Cell Signaling Technology); anti-SQSTM1/p62 rabbit mAb (39749, Cell Signaling Technology); anti-LC3B rabbit mAb (3868, Cell Signaling Technology); anti-GAPDH rabbit mAb (5174, Cell Signaling Technology); anti-NF-κB p65 rabbit mAb (8242, Cell Signaling Technology); anti-NF-κB p65 (Ser536) rabbit mAb (3033, Cell Signaling Technology); and rabbit anti-β-actin (ab227387, Abcam).

### 4.8 Native PAGE

For the native PAGE experiment, cells were lysed in NP-40 buffer (50 mM Tris-HCl, pH 7.4; 150 mM NaCl; 1% NP-40; and 100 µM PMSF). After protein quantification, 15 µg of protein was used for native PAGE. Samples were mixed with 4× native loading buffer (62.5 mM Tris-HCl, pH 6.8; 15% glycerol; 1% deoxycholate; and 0.02% bromophenol blue). Native 7.5% PAGE gels were prepared without SDS. Gels were prerun at 4°C using cathode buffer (25 mM Tris, 192 mM glycine, pH 8.4, and 1% deoxycholate) and anode buffer (25 mM Tris and 192 mM glycine, pH 8.4) in the cathode and anode chambers, respectively, for 30 min at 30 mA. Samples were then electrophoresed at 25 mA and transferred to PVDF membranes for immunoblot analysis.

### 4.9 Immunofluorescence

To verify *Porphyromonas gingivalis* (*P. gingivalis*) infection, HCT116 cells were seeded in culture dishes and cocultured with *P. gingivalis* at an MOI of 100 for 24 h. The treated cells were washed three times with phosphate-buffered saline (PBS) and fixed with 4% paraformaldehyde for 30 min. After being washed again with PBS, the cells were permeabilized with 0.1% Triton X-100 in PBS, blocked with 3% bovine serum albumin (BSA) for 1 h, and incubated with primary antibodies at 4 °C overnight. Following three PBS washes, cells were incubated with fluorophore-conjugated secondary antibodies. Cell membranes were stained with wheat germ agglutinin (WGA), and nuclei were stained with 4′,6-diamidino-2-phenylindole (DAPI). Images were acquired using a Zeiss LSM900 confocal microscope (Carl Zeiss).

### 4.10 Small RNA sequencing and bioinformatic analysis

Total RNA was extracted from large extracellular vesicles (lEVs) derived from uninfected or *P. gingivalis*–infected HCT116 cells using TRIzol^®^ reagent (Life Technologies) according to the manufacturer’s instructions. Small RNAs (18–30 nt) were enriched by polyacrylamide gel electrophoresis (PAGE). Sequencing libraries were constructed using the NEBNext^®^ Small RNA Library Prep Kit (E7300, New England Biolabs) with sequential 3′ and 5′ adapter ligation, reverse transcription, and PCR amplification to generate cDNA libraries. Purified cDNA libraries were quantified and sequenced on an Illumina NovaSeq X Plus platform by Gene Denovo Biotechnology Co. (Guangzhou, China).

Raw sequence files were subjected to quality control analysis. All clean tags were aligned with small RNAs in the GenBank database (Release 209.0) and the Rfam database (Release 11.0) to identify and remove rRNA, scRNA, snoRNA, snRNA, and tRNA. The remaining clean tags were then searched against the miRBase database (Release 22) to identify known miRNAs from the studied species. Unannotated tags were aligned with the ARS-UCD1.2 reference genome. Based on their genomic positions and predicted hairpin structures, novel miRNA candidates were identified using miRDeep2 software (https://github.com/rajewsky-lab/mirdeep2). Differential expression analysis of miRNAs between groups was performed using edgeR software. miRNAs with a fold change ≥ 2 and a *p* value < 0.05 were considered significantly differentially expressed.

### 4.11 Mass spectrometry

For protein extraction and digestion, 30 µg of purified large extracellular vesicle (lEV) protein was used for reduction and alkylation before being loaded onto a filter-aided sample preparation (FASP) membrane. An appropriate amount of trypsin was added for overnight digestion. The digested peptides were then extracted, collected, and spin-dried at low temperature. The dried peptide powder was dissolved in 0.1% formic acid (FA) aqueous solution, and the peptide concentration was determined for label-free quantitative mass spectrometry analysis.

The digested peptides were separated using an EASY-nLC 1200 system. Buffer A consisted of 0.1% FA in water, and Buffer B consisted of 0.1% FA in 80% acetonitrile. First, the loading and analytical columns were equilibrated with 100% Buffer A. The peptides were then transferred to the loading column (C18) using an autosampler and separated on the analytical column at a flow rate of 600 nL/min. The elution gradient was as follows: starting from 6% Buffer B, increasing to 12% after 8 min; then from 13% to 30% over the next 92 min; from 30% to 45% over 10 min; and finally from 45% to 100% within 1 min, followed by incubation with 100% Buffer B for 9 min. The separated peptide samples were analyzed on a Q Exactive HF-X mass spectrometer (Thermo Fisher Scientific) operating in positive ion mode. The full MS scan range was 300–1,400 m/z, with a resolution of 120,000 at 200 m/z, an automatic gain control (AGC) target of 3 × 10^6^, a maximum injection time (IT) of 30 ms, and a dynamic exclusion time of 12 s. For each full MS scan, 60 MS/MS (MS2) spectra were acquired using higher-energy collisional dissociation (HCD) fragmentation with a normalized collision energy of 27%. All identified peptides exhibited mass deviations predominantly within 10 ppm, indicating accurate and reliable identification. The MS2 spectra were subsequently analyzed using the Mascot search engine to obtain scores for each spectrum. The distribution of high peptide scores demonstrated that the mass spectrometry data were of high quality. For qualitative analysis, a stringent peptide false discovery rate (FDR) threshold of ≤0.05 was applied throughout data processing.

### 4.12 Data analysis and bioinformatics

The raw data from mass spectrometry analyses were obtained in RAW format, and the Firmiana data analysis cloud platform was used for qualitative and quantitative analyses. The database search parameters were as follows: enzyme, trypsin; database, human (from NCBI); fixed modification, carbamidomethyl (C); variable modifications, oxidation (M) and acetylation (protein N-terminus); maximum missed cleavages, two; peptide mass tolerance, 20 ppm; and fragment mass tolerance, 50 mmu. The quantified proteomic expression data were normalized to the (−1, 1) interval. The pheatmap R package was then used to classify both the samples and protein expression levels in two dimensions simultaneously (Euclidean distance, average linkage), and a hierarchical clustering heatmap was generated.

### 4.13 Data availability

The raw mass spectrometry data generated in this study have been deposited in the ProteomeXchange Consortium (https://proteomecentral.proteomexchange.org) via the iProX partner repository (https://www.iprox.cn/) (Ma et al., 2019; Chen et al., 2022) with the dataset identifier PXD059913, and PXD068516. The miRNA sequencing data were deposited in the NCBI sequence read archive (http://www.ncbi.nlm.nih.gov/Traces/sra/sra.cgi?view=run_browser) under dataset accession no. SRR34216144, SRR34216143, SRR34216142, SRR34216140, SRR34216141, and SRR34216139.

## Data Availability

The datasets presented in this study can be found in online repositories. The names of the repository/repositories and accession number(s) can be found in the article/**Supplementary Material**.
